# Utility of Fine-Gauge Balloon Catheter for EUS-Guided Hepaticogastrostomy

**DOI:** 10.3390/jcm11195681

**Published:** 2022-09-26

**Authors:** Shin Yagi, Yusuke Kurita, Takamitsu Sato, Sho Hasegawa, Kunihiro Hosono, Noritoshi Kobayashi, Itaru Endo, Yusuke Saigusa, Kensuke Kubota, Atsushi Nakajima

**Affiliations:** 1 Department of Gastroenterology and Hepatology, Yokohama City University School of Medicine, Yokohama 236-0004, Japan; 2 Department of Oncology, School of Medicine, Yokohama City University, Yokohama 236-0004, Japan; 3 Department of Gastroenterological Surgery, Yokohama City University Hospital, Yokohama 236-0004, Japan; 4 Department of Biostatistics, School of Medicine, Yokohama City University, Yokohama 236-0004, Japan

**Keywords:** biliary obstruction, EUS-guided biliary drainage, hepaticogastrostomy, interventional EUS

## Abstract

Background and Purpose: During endoscopic ultrasound-guided hepaticogastrostomy (EUS-HGS), tract dilation is one of the most important steps, and the placement of conventional metal stents with 8.5 Fr delivery devices is difficult due to the large outer shape of the device. Fine-gauge balloon catheters have become popular because of their stricture penetration ability and ease of dilation. This study aimed to evaluate the utility of fine-gauge balloon catheters. Patients and Methods: This retrospective study involved 38 patients who underwent conventional metal stent placement. The patients were classified into two groups: those who underwent dilation with a fine-gauge balloon catheter before initial metal stenting (balloon dilation group) and those who underwent bougie dilation only (non-balloon dilation group). We evaluated the stenting success rate after initial dilation and adverse events. Results: Seventeen and twenty-one patients were included in the balloon dilation and non-balloon dilation groups, respectively. The stenting success rate after initial dilation was 100% (17/17) in the balloon dilation group and 71.4% (15/21) in the non-balloon dilation group (*p* = 0.024). As adverse events, peritonitis was observed in one case (4.8%) in the balloon dilation group, and in three cases (14.3%) in the non-balloon dilation group (*p* = 0.613). Conclusions: Dilation using a fine-gauge balloon catheter before conventional metal stent with 8.5 Fr delivery device placement is considered effective in EUS-HGS.

## 1. Introduction

Endoscopic ultrasound-guided hepaticogastrostomy (EUS-HGS) was developed as an alternative treatment to endoscopic retrograde cholangiopancreatography (ERCP) for biliary obstruction [1,2]. High technical success rates and clinical success rates have been reported for EUS-HGS [3,4,5,6,7]. However, the EUS-HGS procedure consists of several steps, of which tract dilation is one of the most important, because some cases of intrahepatic bile duct and stomach wall dilation are difficult to dilate [8]. In particular, compared with plastic stents, the placement of conventional metal stents with 8.5 Fr delivery devices requires sufficient tract dilation due to the larger outer shape of the insertion device.

Devices for tract dilation are broadly classified into mechanical or electrocautery dilators. Electrocautery dilators have reportedly been effective in dilating the tract [1]. However, electrocautery dilators have been reported to have a high risk of complications such as bleeding; a previous study reported that mechanical dilators are safer and more useful than electrocautery dilators in EUS-HGS [9]. Mechanical dilators are further classified into bougie dilators and balloon dilators. In recent years, fine-gauge balloon catheters (REN biliary dilation catheter; KANEKA, Osaka, Japan) have been introduced into clinical practice and gained popularity due to their ability to penetrate the stricture and dilate the tract. 

The utility of the fine-gauge balloon catheters, compared with bougie dilators, has been unclear in EUS-HGS. Therefore, in this study, we retrospectively investigated the utility of dilation with fine-gauge balloon catheters before the placement of conventional metal stents with 8.5 Fr delivery devices in EUS-HGS compared with bougie dilation.

## 2. Materials and Methods

### 2.1. Patients

This was a retrospective study in which patients who underwent EUS-HGS and successful stent placement at our institution between January 2014 and March 2021 were investigated. EUS-HGS is indicated in our institution when biliary cannulation is not possible, or when access to the duodenal papilla is unfeasible due to surgical anatomical changes or obstruction of the gastric outlet. The inclusion criteria were as follows: (1) mechanical dilation and (2) placement of a self-expandable metal stent (SEMS). Exclusion criteria were as follows: (1) electrocautery dilation, (2) plastic stents (PS), and (3) SEMS with a fine delivery device diameter of 5.9 Fr. This study included EUS-HGS and was not included in endoscopic ultrasound-guided hepaticojejunostomy or endoscopic ultrasound-guided choledochoduodenostomy. Additionally, patients who combined EUS-HGS with antegrade stenting were not included in this study.

This study was approved by the institutional review board of our institution (B200600003). This retrospective, observational study used only medical information and did not compromise the privacy of the participants. All patients received an opt-out form and those who did not consent were excluded.

### 2.2. Dilators

**Figure 1:**
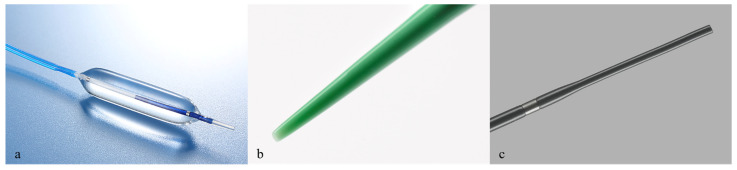
(**a**) shows the fine-gauge balloon catheter (REN biliary dilation catheter; KANEKA, Osaka, Japan). The tip of this balloon catheter is tapered and only 3 Fr wide. This fine-gauge balloon catheter is compatible with 0.025-inch guidewire and is coaxial with guidewire. In this study, catheters with a balloon diameter of 4 mm or 6 mm were used; (**b**) shows an ultra-tapered mechanical (bougie) dilator (ES dilator; Zeon Medical Co., Ltd., Tokyo, Japan). The tip of the ES dilator is 2.5 Fr, and the step between the catheter tip and guidewire is small. The diameter of the ES dilator is 7 Fr; (**c**) shows a bougie dilator (Soehendra Biliary Dilation Catheter; Cook Japan, Tokyo, Japan). A Soehendra Biliary Dilation Catheter with a 4 Fr tip and a 7 Fr catheter diameter was used for initial dilation. A Soehendra Biliary Dilation Catheter with a 6 Fr tip and a 9 Fr catheter diameter was used only for additional dilation.

Figure 1a shows the fine-gauge balloon catheters (REN biliary dilation catheter; KANEKA, Osaka, Japan, 4 mm or 6 mm balloon diameter). The tip of this balloon catheter is tapered with a 3 Fr diameter. This fine-gauge balloon catheter is compatible with 0.025-inch guidewire and is coaxial with guidewire, with a length of 1800 mm. The visual marker in the center of the balloon provides sufficient visibility under fluoroscopy. 

Figure 1b shows an ultra-tapered mechanical (bougie) dilator (ES dilator; Zeon Medical Co., Ltd., Tokyo, Japan). The tip of the ES dilator is 2.5 Fr, and the distance between the catheter tip and guidewire is small. The diameter of the ES dilator is 7 Fr. Figure 1c shows a bougie dilator (Soehendra Biliary Dilation Catheter; Cook Japan, Tokyo, Japan). A Soehendra Biliary Dilation Catheter was used with a 4 Fr tip, 7 Fr catheter diameter, 6 Fr tip and 9 Fr catheter diameter. 

### 2.3. EUS-HGS Procedures

**Figure 2:**
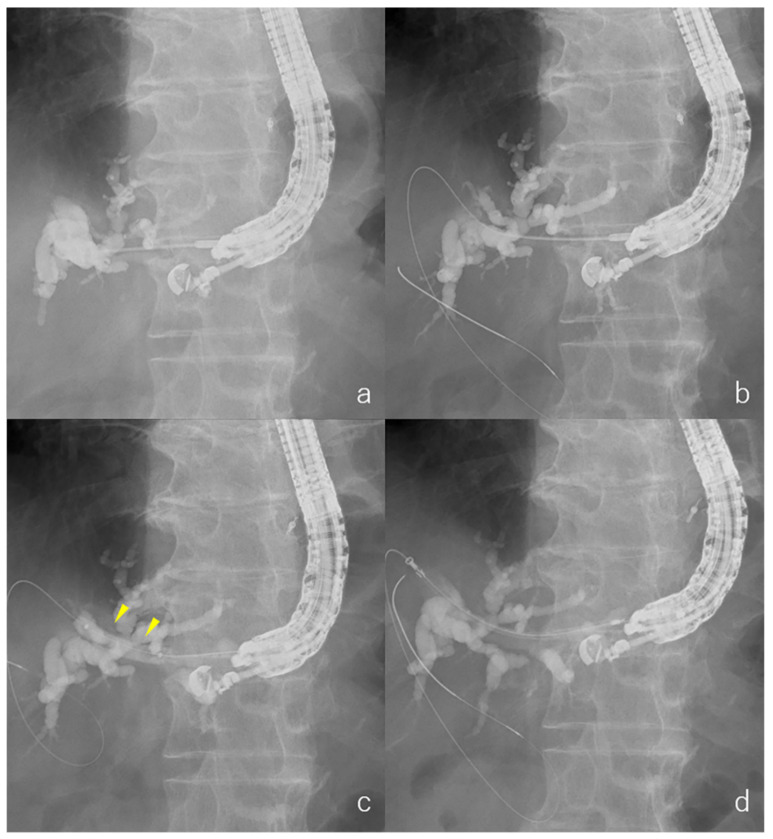
Radiographic images. (**a**) Puncture the bile duct; (**b**) After aspirating bile juice or contrast was used to confirm that it was a bile duct, a guidewire was inserted into the bile duct; (**c**) Tract dilation of a fine-gauge balloon catheter. (**d**) An SEMS was placed.

EUS-HGS was performed in all patients by endoscopists who were trained and experienced in conducting ERCP and EUS. The EUS-HGS procedure in our institution was as follows: Ultrasound endoscopy (EU-ME1; Olympus, Tokyo, Japan and GF-UCT260; Olympus, Tokyo, Japan) was manipulated from inside the stomach to visualize the dilated left intrahepatic bile duct (segment 3: B3, or segment 2: B2). A 19 G needle (EZ shot 3 plus; Olympus, Tokyo, Japan, Sono Tip Pro Control; Medi-Globe GmbH, Rosenheim, Germany or Expect; Boston Scientific, Boston, MA, USA) was used to puncture the bile duct (Figure 2a) without intervening blood vessels using color Doppler ultrasonography. After puncturing the bile duct, at least 2 mL of bile juice was aspirated, or contrast was used to confirm that it was a bile duct. A guidewire (0.025-inch VisiGlide 2 for 19 G needle; Olympus, Tokyo, Japan) was inserted into the bile duct (Figure 2b). Tract dilation was performed using a fine-gauge balloon catheter (Figure 2c), or a bougie dilation catheter alone. A fine-gauge balloon catheter (REN biliary dilation catheter, 4 mm or 6 mm balloon diameter) was used. Tract bougie dilation was performed using a bougie dilation catheter with a 7 Fr diameter (ES dilator or Soehendra Biliary Dilation Catheter).

After tract dilation, an SEMS (6 mm or 8 mm diameter, 12 cm length, 8.5 Fr diameter of the delivery device; Niti-S S-type biliary stent; Taewoong Medical, Seoul, Korea) was placed (Figure 2d). For cases in which SEMS placement was unsuccessful after the initial dilation, additional dilation with a larger-diameter bougie catheter (Soehendra Biliary Dilation Catheter; 9 Fr diameter) or a fine-gauge balloon catheter (REN biliary dilation catheter; 4 mm balloon diameter) was performed. The selection of the type of stent and dilator was dependent on the endoscopist.

### 2.4. Definition of Classification Balloon Dilation Group 

Patients who underwent EUS-HGS and SEMS placement were classified into two groups: those who underwent dilation with the fine-gauge balloon catheter before the initial stent placement (balloon dilation group or BD group) and those who underwent dilation with a bougie dilation catheter only (non-balloon dilation group or non-BD group). A comparative evaluation between the two groups was performed.

### 2.5. Endpoints

The primary endpoint of this study was to compare the stenting success rate after initial dilation between the BD group and non-BD group. The secondary endpoints included adverse events between the BD group and non-BD group. In addition, factors influencing the stenting success rate after initial dilation were assessed in multivariate analysis using logistic regression. Variables used for analysis were biliary obstruction (hilar vs. non hilar), puncture site (B2 vs. B3), diameter of bile duct of puncture site (≥5 mm vs. <5 mm), and type of dilation (BD group vs. non-BD group).

### 2.6. Diagnostic Criteria for Adverse Events

Potential adverse events related to the procedure within 1 month were graded according to the severity grading system of the American Society for Gastrointestinal Endoscopy lexicon [10]. Peritonitis was diagnosed if, in addition to abdominal pain, fever or elevated inflammatory markers on blood examination were observed within 1 day after EUS-HGS. Bile leakage around the HGS stent on CT scan the day after EUS-HGS was diagnosed as biloma [11].

### 2.7. Statistical Analysis

The proportions of categorical variables were compared using Fisher’s exact tests. The distribution of continuous variables in the cohorts were compared using the Mann–Whitney U test. Statistical significance was set at *p* < 0.05. These statistical analyses were performed using SPSS version 27 software (SPSS, version 27; IBM, Armonk, NY, USA). In multivariate analyses, Firth logistic regression analysis was performed to assess the factors influencing the stenting success rate after initial dilation using R version 4.1.0 (R, version 4.1.0; Foundation for Statistical Computing, Vienna, Austria).

## 3. Results

**Figure 3:**
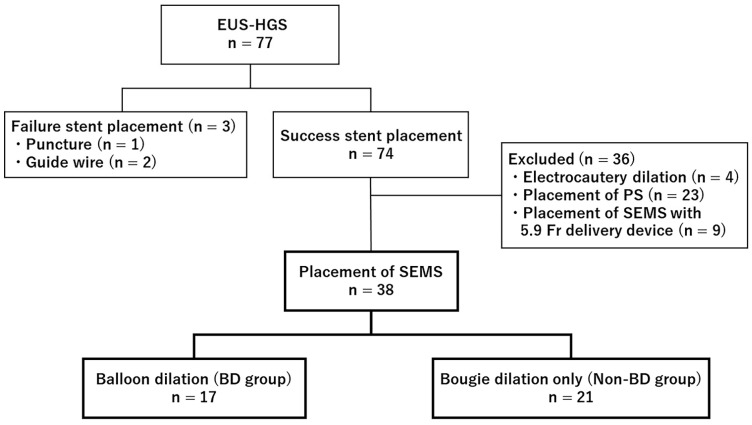
Flow diagram showing the number of patients.

The flowchart of this study is shown in Figure 3. During this study period, EUS-HGS was performed on 77 patients, and it was unsuccessful in 3 patients. The causes of failure were puncture in 1 patient and wire guiding in 2 patients. There were 4 cases of electrocautery dilation, 23 cases of placement of plastic stents, and 9 cases of placement of SEMS with a fine delivery device diameter of 5.9 Fr, all of which were excluded. Among the 38 patients with SEMS with a delivery device diameter of 8.5 Fr who were included in the study, 17 were in the BD group and 21 were in the non-BD group. Patient characteristics are presented in Table 1. There were no statistically significant differences in age, sex, etiology or location of biliary obstruction, or biliary drainage before EUS-HGS between the groups.

### 3.1. Outcomes of EUS-HGS

The outcomes of EUS-HGS between the BD group and non-BD group are shown in Table 2. In the BD group, 82.4% (14/17) used 4 mm diameter and 17.6% (3/17) used 6 mm diameter fine-gauge balloon catheters for initial dilation. In the non-BD group, 85.7% (18/21) used ES dilator (7 Fr diameter) and 14.3% (3/21) used Soehendra Biliary Dilation Catheters (7 Fr diameter) for initial dilation. The stenting success rate after initial dilation was 100% (17/17) in the BD group and 71.4% (15/21) in the non-BD group (*p* = 0.024). 

In the non-BD group, six patients had failed SEMS placement after the initial tract dilation. Of the six cases that required additional dilation, four required additional dilation with a fine-gauge balloon catheter (4 mm diameter), and two required additional dilation with Soehendra Biliary Dilation Catheters (9 Fr diameter). 

### 3.2. Adverse Events

Concerning adverse events, peritonitis was observed in one case (5.9%) in the BD group and in three cases (14.3%) in the non-BD group. Biloma occurred in one case (4.8%) in the non-BD group only. However, there was no significant difference between the BD group and non-BD group in the incidence of adverse events (Table 2). All patients with adverse events improved within a few days after the procedures with conservative treatment. No bleeding or other serious complications were observed.

### 3.3. Factors Influencing the Stenting Success Rate after Initial Dilation

The only factor that influenced the stenting success rate after initial dilation was the type of dilation (BD group vs. non-BD group) (Table 3). Although the puncture site (B2 vs. B3) was not a significant factor influencing the stenting success rate after initial dilation, the cases in which SEMS placement failed after the initial tract dilation were all B3 bile ducts. All cases in which the B2 bile duct was punctured were successful in the placement of the SEMS after initial tract dilation, regardless of the dilatation method.

## 4. Discussion

EUS-HGS has gained popularity as an alternative biliary drainage method in recent years. Various techniques for tract dilation have been developed using EUS-HGS. 

In the present study, we retrospectively evaluated the utility of a fine-gauge balloon catheter compared with bougie dilators in EUS-HGS. The stenting success rate after initial dilation was significantly better in the BD group than in the non-BD group. In all cases of unsuccessful SEMS placement after the initial tract dilation in the non-BD group, SEMS placement was possible after additional dilation with a fine-gauge balloon catheter. Therefore, tract dilation with a fine-gauge balloon catheter prior to SEMS with an 8.5 Fr delivery device placement may be more effective than bougie dilation alone in EUS-HGS. 

The statistically significant factor that influenced the stenting success rate after initial dilation was the type of dilation only (BD group vs. non-BD group). Although there was no significant difference in the puncture site (B2 vs. B3), all cases of failed SEMS placement after initial tract dilation were cases in which the B3 bile duct was punctured. In contrast, in all cases in which the B2 bile duct was punctured, SEMS was successfully placed regardless of the dilation method. If the B2 bile duct is punctured, there is a risk that the puncture site at the intraluminal portion might be from the esophagus [12]. However, the B2 bile duct runs on a relatively straight line between the endoscopic ultrasound and the puncture line; therefore, the procedure of stenting may be easier. The results of the present study suggest that balloon dilation may not be necessary in EUS-HGS in cases of puncture to the B2 bile duct, before metal stenting. On the other hand, the B3 bile duct exhibits a tight angle between the ultrasound endoscope and the puncture line, which may have made the insertion of the device difficult. Metal stent placement of the B3 bile duct was possible in all patients after balloon dilation in this study. Therefore, EUS-HGS for the B3 bile duct may require balloon dilation. 

In the present study, one case of peritonitis (5.9%) was observed in the BD group, and three cases of peritonitis (14.3%) and one case of biloma were observed in the non-BD group, as adverse events. Bile peritonitis has been reported to occur frequently in EUS-HGS [11]. In a previous report that retrospectively evaluated bile leakage during ultrasound endoscopy-guided biliary drainage (EUS-BD) for malignant biliary obstruction in a multicenter setting, bile leakage was more frequently observed in patients with plastic stent placement compared with those with covered metal stent placement. They concluded that the use of covered metal stents is a strategy to prevent bile leakage [13]. On the other hand, another report suggests that bile leakage can occur even when covered metal stents are used, and that the cause is because during the EUS-HGS procedure, the exchange of various devices is needed before stent placement [12]. In the present study, two of six patients who underwent additional dilation in the non-BD group had peritonitis (33.3%), and two of thirty-two patients who did not have additional dilation had peritonitis (6.3%) (*p* = 0.11). Although there was no significant difference, this suggests that bile may have leaked from the tract due to the need for device exchange for additional dilation and treatment.

This study had certain limitations. First, this was a retrospective study, and there were no clear criteria for the selection of stents or dilators, which depended on the endoscopist. Second, this was a single-center study with a small number of cases. Factors influencing the stenting success rate after initial dilation were assessed in multivariate analysis, but the number of cases was small and multivariate analysis of a larger number of cases is desirable. Further prospective randomized controlled trials with a sufficient number of patients are being considered to confirm the results of this study. Third, we excluded cases of SEMS placement with the recently developed 5.9 Fr delivery device. Insertion of SEMS with 5.9 Fr delivery devices may be easy and may not require balloon dilation. We are considering a study including SEMS with a 5.9 Fr delivery device.

## 5. Conclusions

In conclusion, dilation using a fine-gauge balloon catheter prior to conventional SEMS with an 8.5 Fr delivery device placement is considered effective in EUS-HGS, especially when puncturing the B3 bile duct.

## Figures and Tables

**Table 1:** Patient characteristics.

	Total	BD Group	Non-BD Group	*p*
No. of cases (%)	*n* = 38	*n* = 17	*n* = 21	
Age, years, median (range)	69 (36–84)	69 (56–83)	69 (36–84)	0.685
Sex (%)				0.506
Male	24 (63.2%)	12 (70.6%)	12 (57.1%)	
Female	14 (36.8%)	5 (29.4%)	9 (42.9%)	
Etiology of biliary obstruction (%)				0.259
Pancreatic cancer	18 (47.4%)	10 (58.8%)	8 (38.1%)	
Biliary cancer	9 (23.7%)	2 (11.8%)	7 (33.3%)	
Other	11 (28.9%)	5 (29.4%)	6 (28.6%)	
Biliary obstruction (%)				0.062
Distal	26 (68.4%)	12 (70.6%)	14 (66.7%)	
Hilar	9 (23.7%)	2 (11.8%)	7 (33.3%)	
Postoperative anastomosis	3 (7.9%)	3 (17.6%)	0	

BD group: balloon dilation group, Non-BD group: non-balloon dilation group, EUS-2 HGS: endoscopic ultrasound-guided hepaticogastrostomy.

**Table 2:** Outcomes of EUS-HGS between BD groups or non-BD groups.

	Total	BD Group	Non-BD Group	*p*
No. of cases (%)	*n* = 38	*n* = 17	*n* = 21	
Procedure time, min, median (range)	35.5 (17–80)	37 (19–80)	32 (17–60)	0.601
Diameter of bile duct of puncture site, mm, median (range)	6 (4–15)	5.8 (4–9)	6 (4–15)	0.149
Puncture site (%)				0.197
B2	6 (15.8%)	1 (5.9%)	5 (23.8%)	
B3	32 (84.2%)	16 (94.1%)	16 (76.2%)	
Initial dilation (%)				-
Balloon dilator				
REN 4 mm diameter	14 (36.8%)	14 (82.4%)		
REN 6 mm diameter	3 (7.9%)	3 (17.6%)		
Bougie dilator				
ES dilator (7 Fr)	13 (34.2%)		13 (85.7%)	
Soehendra (7 Fr)	3 (7.9%)		3 (14.3%)	
Stenting success rate after initial dilation (%)	84.2% (32/38)	100% (17/17)	71.4% (15/21)	0.024
Additional dilation (%)	6 (15.8%)	0	6 (28.6%)	0.024
Balloon dilator				
REN 4 mm diameter	4 (10.5%)	0	4 (19.0%)	
Bougie dilator				
Soehendra (9 Fr)	2 (5.3%)	0	2 (9.5%)	
Adverse events (%)				
Peritonitis	4 (10.5%)	1 (5.9%)	3 (14.3%)	0.613
Biloma	1 (2.6%)	0	1 (4.8%)	1.000
Bleeding	0	0	0	-
Stent migration	0	0	0	-

EUS-HGS: EUS-guided hepaticogastrostomy, BD group: balloon dilation group, non-BD group: non-balloon dilation group, B2: bile duct of segment 2 of the liver, B3: bile duct of segment 3 of the liver, SEMS: self-expandable metal stent.

**Table 3:** Multivariate analysis of factors influencing the stenting success rate after initial dilation.

Variable		Stenting Success Rate after Initial Dilation (%)	OR	95% CI	*p*
Biliary obstruction	Hilar	77.8% (7/9)	0.266	0.022–1.991	0.202
	Non hilar	86.2% (25/29)			
Puncture site	B3	79.5% (26/32)	0.109	0.0006–1.539	0.114
	B2	100.0% (6/6)			
Diameter of bile duct of puncture site	≥5 mm	83.3% (25/30)	0.440	0.023–3.952	0.487
	<5 mm	87.5% (7/8)			
Type of dilation	Non-BD group	71.4% (15/21)	0.060	0.0004–0.657	0.013
	BD group	100.0% (17/17)			

OR: odds ratio, CI: confidence interval, BD group: balloon dilation group, non-BD group: non-balloon dilation group, B2: bile duct of segment 2 of the liver, B3: bile duct of segment 3 of the liver, SEMS: self-expandable metal stent.

## Data Availability

Data are available on request because of restrictions, e.g., privacy or ethics.
